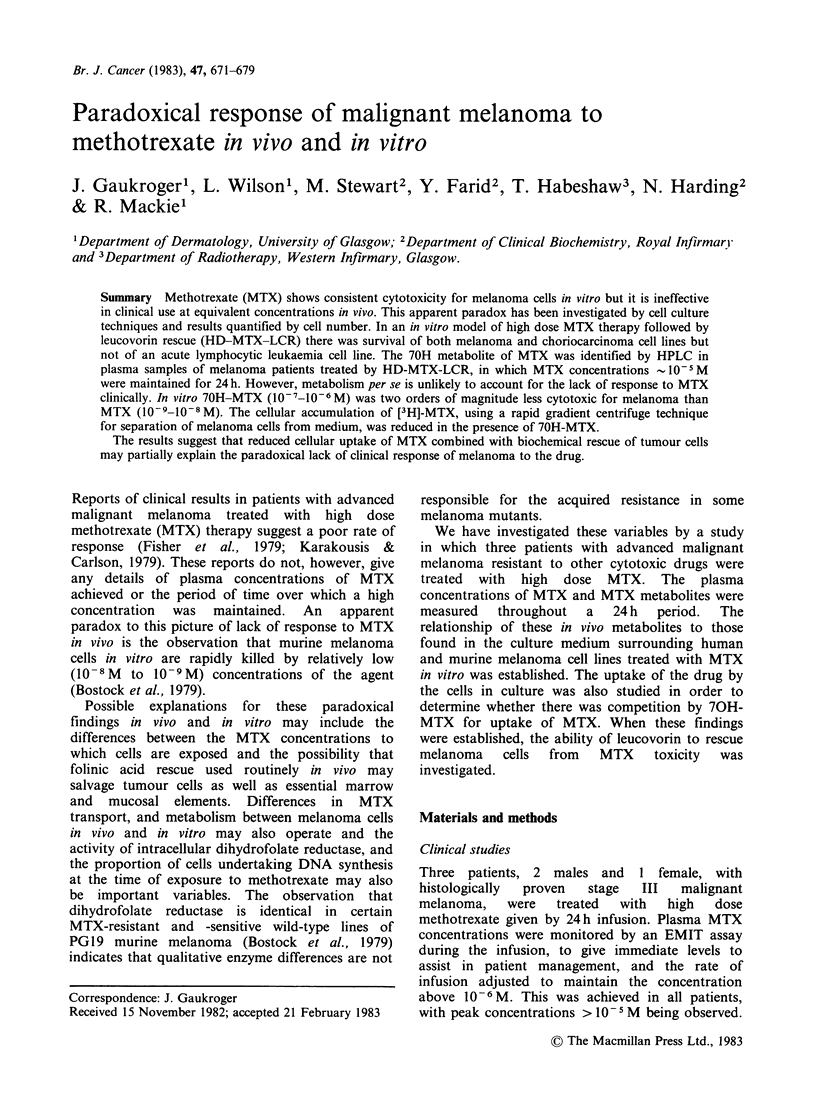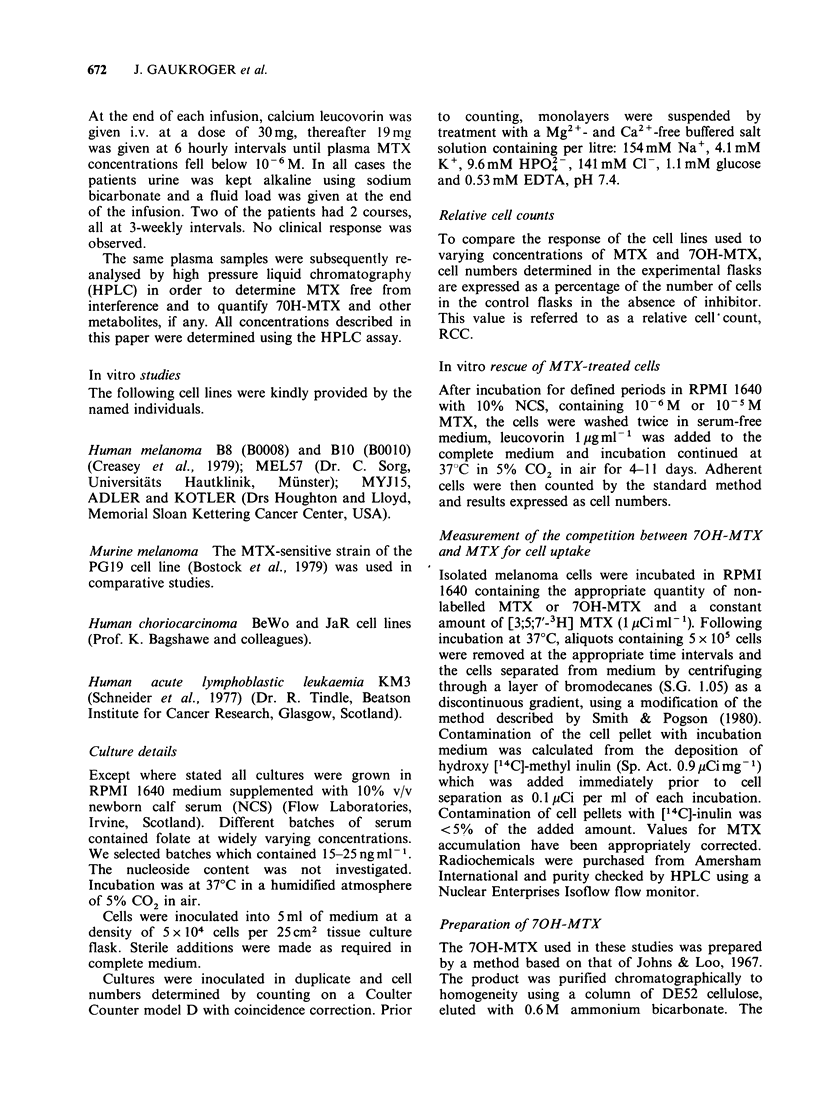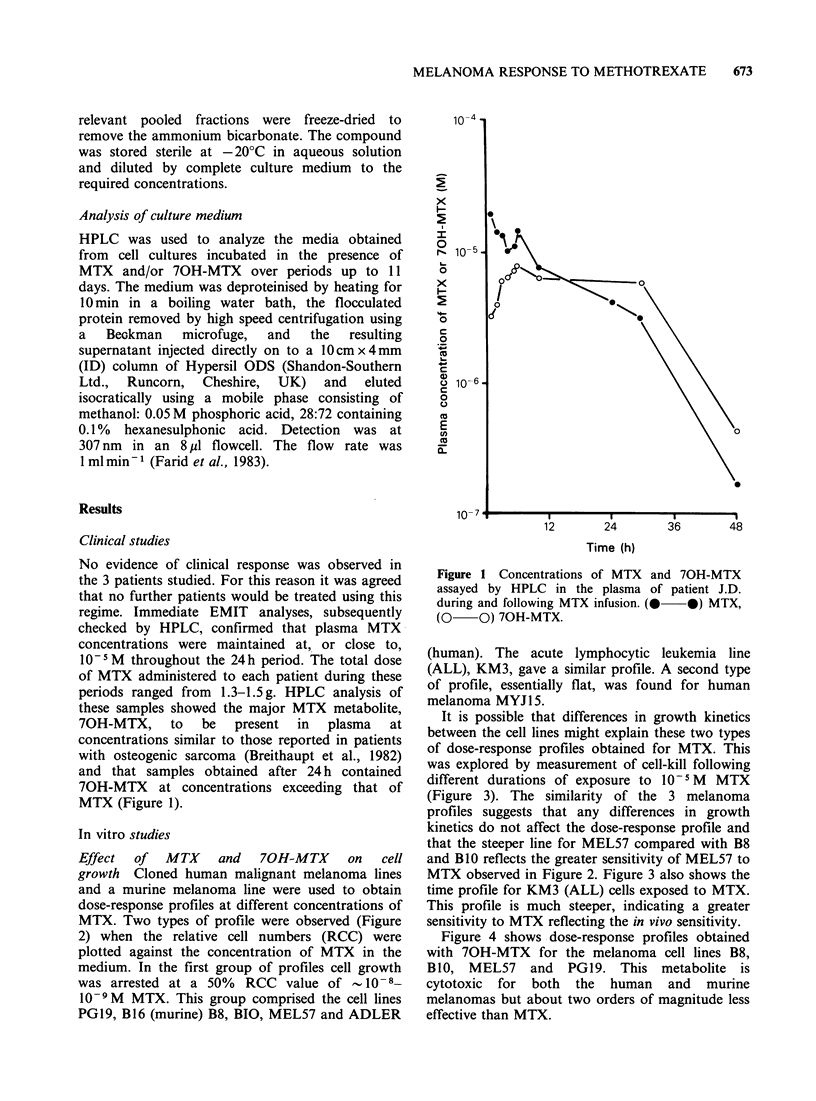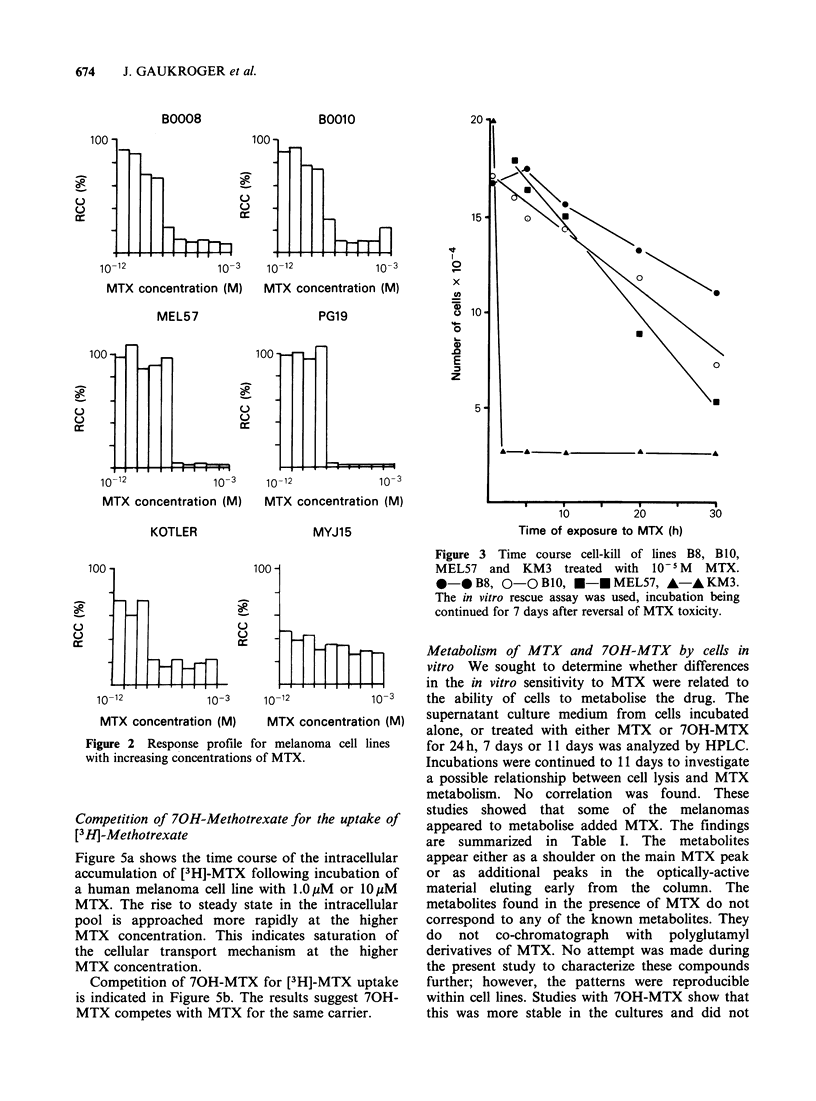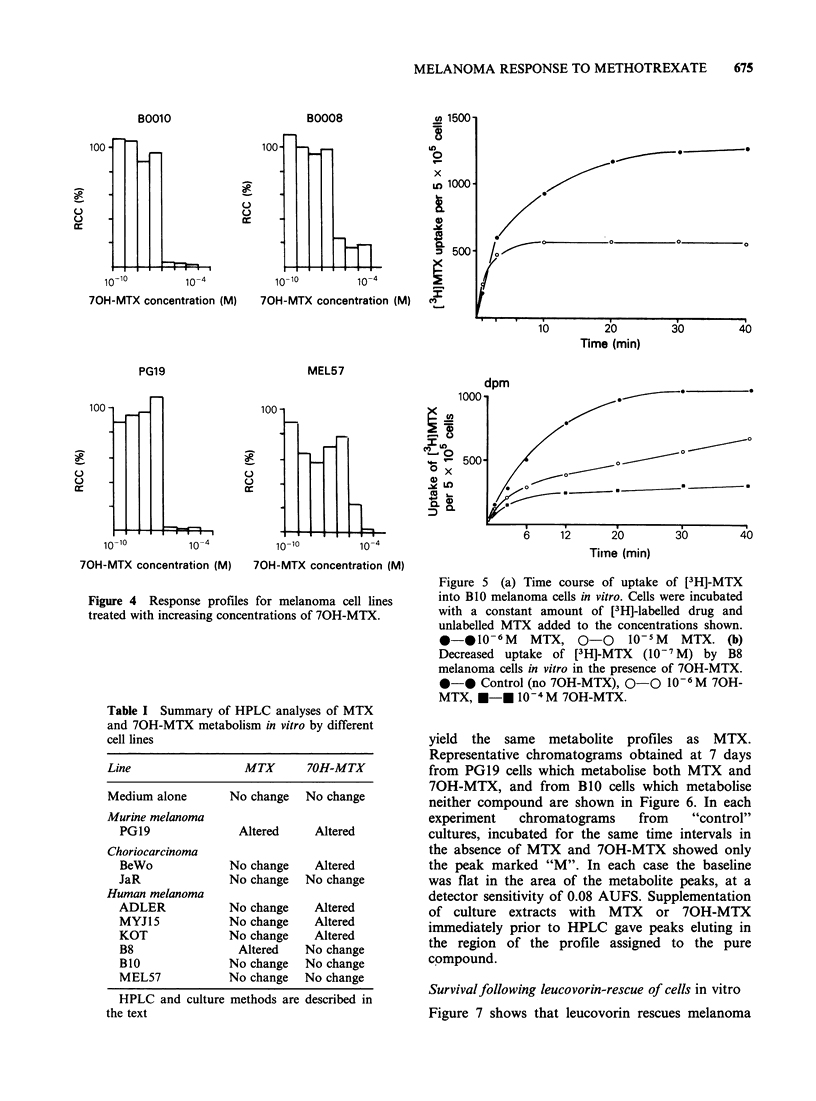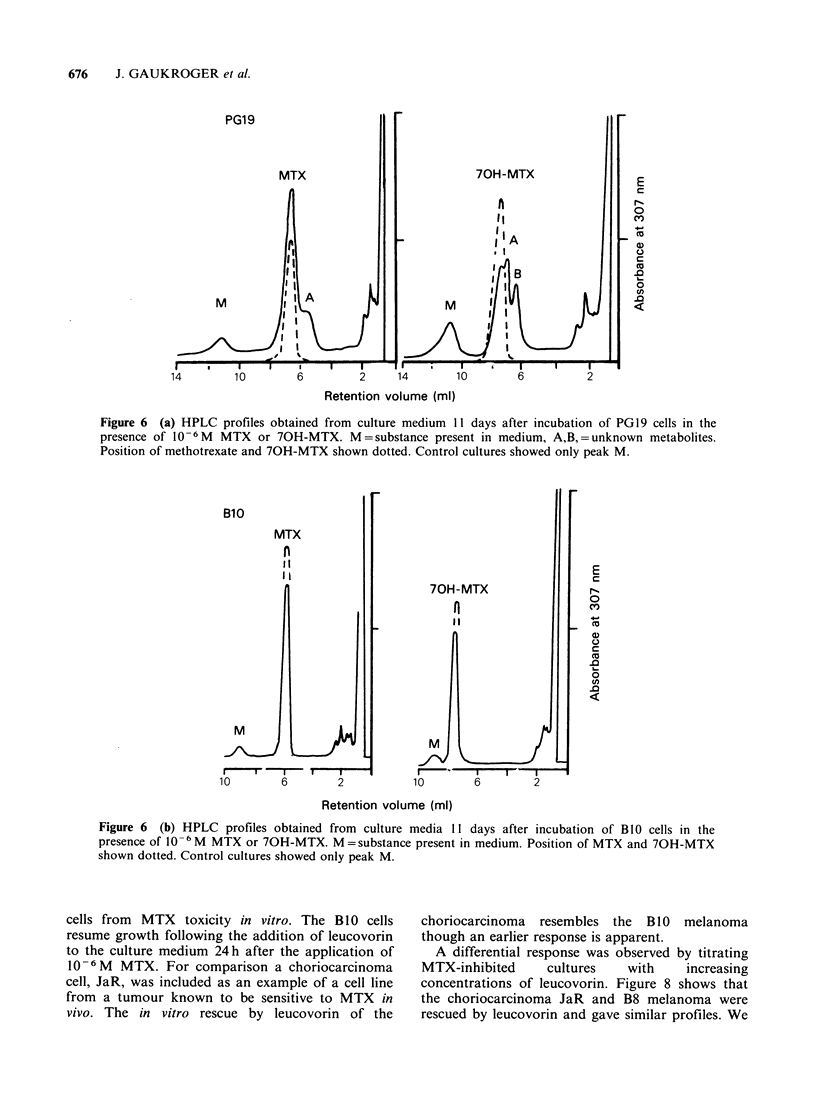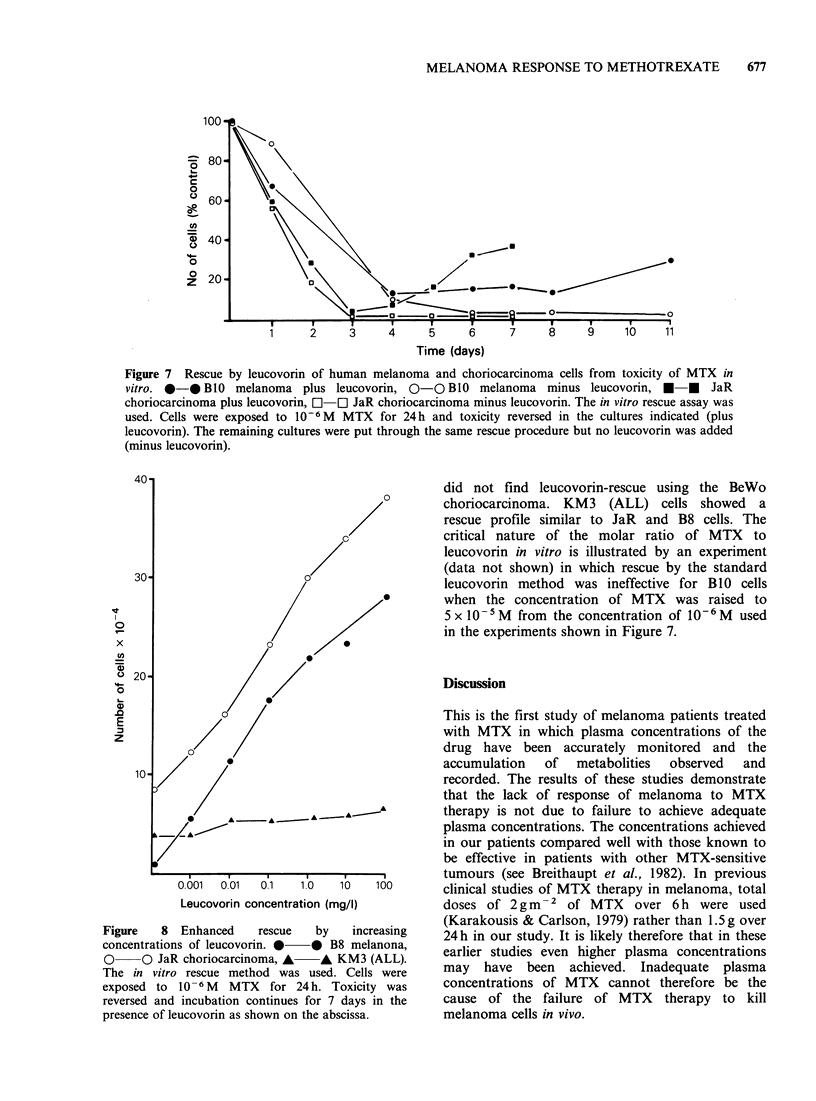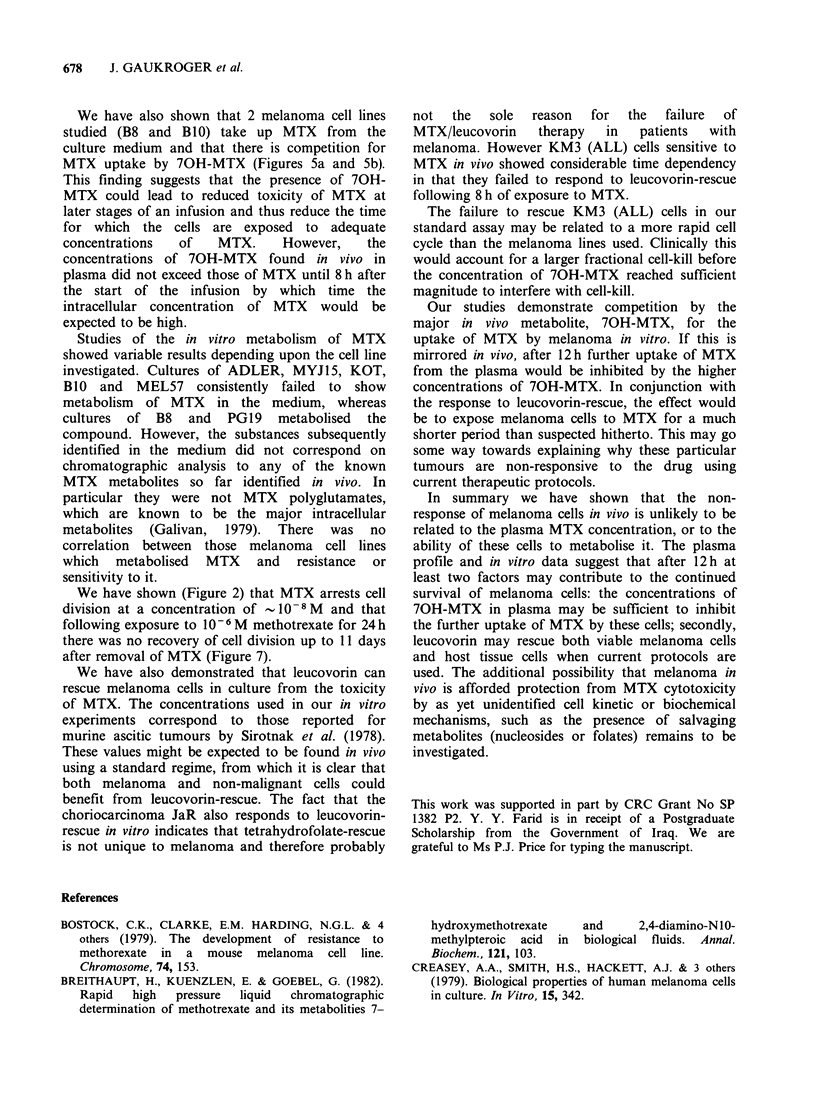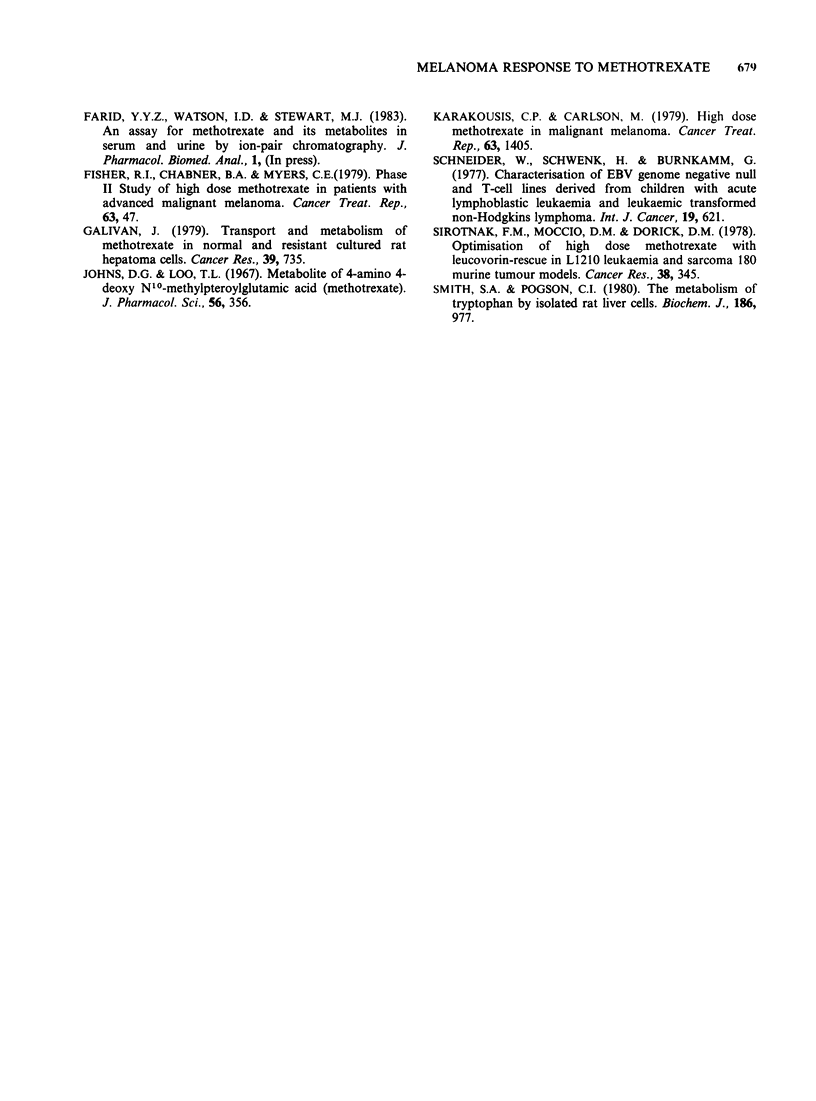# Paradoxical response of malignant melanoma to methotrexate in vivo and in vitro.

**DOI:** 10.1038/bjc.1983.105

**Published:** 1983-05

**Authors:** J. Gaukroger, L. Wilson, M. Stewart, Y. Farid, T. Habeshaw, N. Harding, R. Mackie

## Abstract

Methotrexate (MTX) shows consistent cytotoxicity for melanoma cells in vitro but it is ineffective in clinical use at equivalent concentrations in vivo. This apparent paradox has been investigated by cell culture techniques and results quantified by cell number. In an in vitro model of high dose MTX therapy followed by leucovorin rescue (HD-MTX-LCR) there was survival of both melanoma and choriocarcinoma cell lines but not of an acute lymphocytic leukaemia cell line. The 70H metabolite of MTX was identified by HPLC in plasma samples of melanoma patients treated by HD-MTX-LCR, in which MTX concentrations approximately 10(-5) M were maintained for 24 h. However, metabolism per se is unlikely to account for the lack of response to MTX clinically. In vitro 70H MTX (10(-7) - 10(-6) M) was two orders of magnitude less cytotoxic for melanoma than MTX (10(-9) - 10(-8) M). The cellular accumulation of [3H]-MTX, using a rapid gradient centrifuge technique for separation of melanoma cells from medium, was reduced in the presence of 70H-MTX. The results suggest that reduced cellular uptake of MTX combined with biochemical rescue of tumour cells may partially explain the paradoxical lack of clinical response of melanoma to the drug.


*Br. J. Cancer* (1983), **47**, 671–679

### Paradoxical response of malignant melanoma to methotrexate *in vivo* and *in vitro*

J. Gaukroger[1], L. Wilson[1], M. Stewart[2], Y. Farid[2], T. Habeshaw[3], N. Harding[2] & R. Mackie[1]

[1]*Department of Dermatology, University of Glasgow;* [2]*Department of Clinical Biochemistry, Royal Infirmary and* [3]*Department of Radiotherapy, Western Infirmary, Glasgow.*

**Summary** Methotrexate (MTX) shows consistent cytotoxicity for melanoma cells *in vitro* but it is ineffective in clinical use at equivalent concentrations *in vivo*. This apparent paradox has been investigated by cell culture techniques and results quantified by cell number. In an *in vitro* model of high dose MTX therapy followed by leucovorin rescue (HD–MTX–LCR) there was survival of both melanoma and choriocarcinoma cell lines but not of an acute lymphocytic leukaemia cell line. The 70H metabolite of MTX was identified by HPLC in plasma samples of melanoma patients treated by HD-MTX-LCR, in which MTX concentrations $\sim 10^{-5}$ M were maintained for 24 h. However, metabolism *per se* is unlikely to account for the lack of response to MTX clinically. *In vitro* 70H–MTX ($10^{-7}$–$10^{-6}$ M) was two orders of magnitude less cytotoxic for melanoma than MTX ($10^{-9}$–$10^{-8}$ M). The cellular accumulation of [$^3$H]-MTX, using a rapid gradient centrifuge technique for separation of melanoma cells from medium, was reduced in the presence of 70H-MTX.

The results suggest that reduced cellular uptake of MTX combined with biochemical rescue of tumour cells may partially explain the paradoxical lack of clinical response of melanoma to the drug.

Reports of clinical results in patients with advanced malignant melanoma treated with high dose methotrexate (MTX) therapy suggest a poor rate of response (Fisher *et al.*, 1979; Karakousis & Carlson, 1979). These reports do not, however, give any details of plasma concentrations of MTX achieved or the period of time over which a high concentration was maintained. An apparent paradox to this picture of lack of response to MTX *in vivo* is the observation that murine melanoma cells *in vitro* are rapidly killed by relatively low ($10^{-8}$ M to $10^{-9}$ M) concentrations of the agent (Bostock *et al.*, 1979).

Possible explanations for these paradoxical findings *in vivo* and *in vitro* may include the differences between the MTX concentrations to which cells are exposed and the possibility that folinic acid rescue used routinely *in vivo* may salvage tumour cells as well as essential marrow and mucosal elements. Differences in MTX transport, and metabolism between melanoma cells *in vivo* and *in vitro* may also operate and the activity of intracellular dihydrofolate reductase, and the proportion of cells undertaking DNA synthesis at the time of exposure to methotrexate may also be important variables. The observation that dihydrofolate reductase is identical in certain MTX-resistant and -sensitive wild-type lines of PG19 murine melanoma (Bostock *et al.*, 1979) indicates that qualitative enzyme differences are not responsible for the acquired resistance in some melanoma mutants.

We have investigated these variables by a study in which three patients with advanced malignant melanoma resistant to other cytotoxic drugs were treated with high dose MTX. The plasma concentrations of MTX and MTX metabolites were measured throughout a 24 h period. The relationship of these *in vivo* metabolites to those found in the culture medium surrounding human and murine melanoma cell lines treated with MTX *in vitro* was established. The uptake of the drug by the cells in culture was also studied in order to determine whether there was competition by 7OH-MTX for uptake of MTX. When these findings were established, the ability of leucovorin to rescue melanoma cells from MTX toxicity was investigated.

### Materials and methods

### *Clinical studies*

Three patients, 2 males and 1 female, with histologically proven stage III malignant melanoma, were treated with high dose methotrexate given by 24 h infusion. Plasma MTX concentrations were monitored by an EMIT assay during the infusion, to give immediate levels to assist in patient management, and the rate of infusion adjusted to maintain the concentration above $10^{-6}$ M. This was achieved in all patients, with peak concentrations $>10^{-5}$ M being observed.

Correspondence: J. Gaukroger
Received 15 November 1982; accepted 21 February 1983

© The Macmillan Press Ltd., 1983

At the end of each infusion, calcium leucovorin was given i.v. at a dose of 30 mg, thereafter 19 mg was given at 6 hourly intervals until plasma MTX concentrations fell below $10^{-6}$ M. In all cases the patients urine was kept alkaline using sodium bicarbonate and a fluid load was given at the end of the infusion. Two of the patients had 2 courses, all at 3-weekly intervals. No clinical response was observed.

The same plasma samples were subsequently re-analysed by high pressure liquid chromatography (HPLC) in order to determine MTX free from interference and to quantify 7OH-MTX and other metabolites, if any. All concentrations described in this paper were determined using the HPLC assay.

### In vitro *studies*

The following cell lines were kindly provided by the named individuals.

*Human melanoma* B8 (B0008) and B10 (B0010) (Creasey *et al.*, 1979); MEL57 (Dr. C. Sorg, Universitäts Hautklinik, Münster); MYJ15, ADLER and KOTLER (Drs Houghton and Lloyd, Memorial Sloan Kettering Cancer Center, USA).

*Murine melanoma* The MTX-sensitive strain of the PG19 cell line (Bostock *et al.*, 1979) was used in comparative studies.

*Human choriocarcinoma* BeWo and JaR cell lines (Prof. K. Bagshawe and colleagues).

*Human acute lymphoblastic leukaemia* KM3 (Schneider *et al.*, 1977) (Dr. R. Tindle, Beatson Institute for Cancer Research, Glasgow, Scotland).

### *Culture details*

Except where stated all cultures were grown in RPMI 1640 medium supplemented with 10% v/v newborn calf serum (NCS) (Flow Laboratories, Irvine, Scotland). Different batches of serum contained folate at widely varying concentrations. We selected batches which contained 15–25 ng ml$^{-1}$. The nucleoside content was not investigated. Incubation was at 37°C in a humidified atmosphere of 5% $CO_2$ in air.

Cells were inoculated into 5 ml of medium at a density of $5 \times 10^4$ cells per 25 cm$^2$ tissue culture flask. Sterile additions were made as required in complete medium.

Cultures were inoculated in duplicate and cell numbers determined by counting on a Coulter Counter model D with coincidence correction. Prior to counting, monolayers were suspended by treatment with a $Mg^{2+}$- and $Ca^{2+}$-free buffered salt solution containing per litre: 154 mM $Na^+$, 4.1 mM $K^+$, 9.6 mM $HPO_4^{2-}$, 141 mM $Cl^-$, 1.1 mM glucose and 0.53 mM EDTA, pH 7.4.

### *Relative cell counts*

To compare the response of the cell lines used to varying concentrations of MTX and 7OH-MTX, cell numbers determined in the experimental flasks are expressed as a percentage of the number of cells in the control flasks in the absence of inhibitor. This value is referred to as a relative cell count, RCC.

### In vitro *rescue of MTX-treated cells*

After incubation for defined periods in RPMI 1640 with 10% NCS, containing $10^{-6}$ M or $10^{-5}$ M MTX, the cells were washed twice in serum-free medium, leucovorin 1 μg ml$^{-1}$ was added to the complete medium and incubation continued at 37°C in 5% $CO_2$ in air for 4–11 days. Adherent cells were then counted by the standard method and results expressed as cell numbers.

### *Measurement of the competition between 7OH-MTX and MTX for cell uptake*

Isolated melanoma cells were incubated in RPMI 1640 containing the appropriate quantity of non-labelled MTX or 7OH-MTX and a constant amount of [3;5;7′-$^3$H] MTX (1 μCi ml$^{-1}$). Following incubation at 37°C, aliquots containing $5 \times 10^5$ cells were removed at the appropriate time intervals and the cells separated from medium by centrifuging through a layer of bromodecanes (S.G. 1.05) as a discontinuous gradient, using a modification of the method described by Smith & Pogson (1980). Contamination of the cell pellet with incubation medium was calculated from the deposition of hydroxy [$^{14}$C]-methyl inulin (Sp. Act. 0.9 μCi mg$^{-1}$) which was added immediately prior to cell separation as 0.1 μCi per ml of each incubation. Contamination of cell pellets with [$^{14}$C]-inulin was <5% of the added amount. Values for MTX accumulation have been appropriately corrected. Radiochemicals were purchased from Amersham International and purity checked by HPLC using a Nuclear Enterprises Isoflow flow monitor.

### *Preparation of 7OH-MTX*

The 7OH-MTX used in these studies was prepared by a method based on that of Johns & Loo, 1967. The product was purified chromatographically to homogeneity using a column of DE52 cellulose, eluted with 0.6 M ammonium bicarbonate. The relevant pooled fractions were freeze-dried to remove the ammonium bicarbonate. The compound was stored sterile at −20°C in aqueous solution and diluted by complete culture medium to the required concentrations.

*Analysis of culture medium*

HPLC was used to analyze the media obtained from cell cultures incubated in the presence of MTX and/or 7OH-MTX over periods up to 11 days. The medium was deproteinised by heating for 10 min in a boiling water bath, the flocculated protein removed by high speed centrifugation using a Beckman microfuge, and the resulting supernatant injected directly on to a 10 cm × 4 mm (ID) column of Hypersil ODS (Shandon-Southern Ltd., Runcorn, Cheshire, UK) and eluted isocratically using a mobile phase consisting of methanol: 0.05 M phosphoric acid, 28:72 containing 0.1% hexanesulphonic acid. Detection was at 307 nm in an 8 μl flowcell. The flow rate was 1 ml min$^{-1}$ (Farid *et al.*, 1983).

### Results

*Clinical studies*

No evidence of clinical response was observed in the 3 patients studied. For this reason it was agreed that no further patients would be treated using this regime. Immediate EMIT analyses, subsequently checked by HPLC, confirmed that plasma MTX concentrations were maintained at, or close to, $10^{-5}$ M throughout the 24 h period. The total dose of MTX administered to each patient during these periods ranged from 1.3–1.5 g. HPLC analysis of these samples showed the major MTX metabolite, 7OH-MTX, to be present in plasma at concentrations similar to those reported in patients with osteogenic sarcoma (Breithaupt et al., 1982) and that samples obtained after 24 h contained 7OH-MTX at concentrations exceeding that of MTX (Figure 1).

In vitro *studies*

*Effect of MTX and 7OH-MTX on cell growth* Cloned human malignant melanoma lines and a murine melanoma line were used to obtain dose-response profiles at different concentrations of MTX. Two types of profile were observed (Figure 2) when the relative cell numbers (RCC) were plotted against the concentration of MTX in the medium. In the first group of profiles cell growth was arrested at a 50% RCC value of ~$10^{-8}$–$10^{-9}$ M MTX. This group comprised the cell lines PG19, B16 (murine) B8, BIO, MEL57 and ADLER

**Figure 1** Concentrations of MTX and 7OH-MTX assayed by HPLC in the plasma of patient J.D. during and following MTX infusion. (●——●) MTX, (○——○) 7OH-MTX.

(human). The acute lymphocytic leukemia line (ALL), KM3, gave a similar profile. A second type of profile, essentially flat, was found for human melanoma MYJ15.

It is possible that differences in growth kinetics between the cell lines might explain these two types of dose-response profiles obtained for MTX. This was explored by measurement of cell-kill following different durations of exposure to $10^{-5}$ M MTX (Figure 3). The similarity of the 3 melanoma profiles suggests that any differences in growth kinetics do not affect the dose-response profile and that the steeper line for MEL57 compared with B8 and B10 reflects the greater sensitivity of MEL57 to MTX observed in Figure 2. Figure 3 also shows the time profile for KM3 (ALL) cells exposed to MTX. This profile is much steeper, indicating a greater sensitivity to MTX reflecting the *in vivo* sensitivity.

Figure 4 shows dose-response profiles obtained with 7OH-MTX for the melanoma cell lines B8, B10, MEL57 and PG19. This metabolite is cytotoxic for both the human and murine melanomas but about two orders of magnitude less effective than MTX.

**Figure 2** Response profile for melanoma cell lines with increasing concentrations of MTX.

**Figure 3** Time course cell-kill of lines B8, B10, MEL57 and KM3 treated with $10^{-5}$ M MTX. ●—● B8, ○—○ B10, ■—■ MEL57, ▲—▲ KM3. The *in vitro* rescue assay was used, incubation being continued for 7 days after reversal of MTX toxicity.

*Competition of 7OH-Methotrexate for the uptake of [³H]-Methotrexate*

Figure 5a shows the time course of the intracellular accumulation of [$^3$H]-MTX following incubation of a human melanoma cell line with 1.0 μM or 10 μM MTX. The rise to steady state in the intracellular pool is approached more rapidly at the higher MTX concentration. This indicates saturation of the cellular transport mechanism at the higher MTX concentration.

Competition of 7OH-MTX for [$^3$H]-MTX uptake is indicated in Figure 5b. The results suggest 7OH-MTX competes with MTX for the same carrier.

*Metabolism of MTX and 7OH-MTX by cells in vitro* We sought to determine whether differences in the *in vitro* sensitivity to MTX were related to the ability of cells to metabolise the drug. The supernatant culture medium from cells incubated alone, or treated with either MTX or 7OH-MTX for 24 h, 7 days or 11 days was analyzed by HPLC. Incubations were continued to 11 days to investigate a possible relationship between cell lysis and MTX metabolism. No correlation was found. These studies showed that some of the melanomas appeared to metabolise added MTX. The findings are summarized in Table I. The metabolites appear either as a shoulder on the main MTX peak or as additional peaks in the optically-active material eluting early from the column. The metabolites found in the presence of MTX do not correspond to any of the known metabolites. They do not co-chromatograph with polyglutamyl derivatives of MTX. No attempt was made during the present study to characterize these compounds further; however, the patterns were reproducible within cell lines. Studies with 7OH-MTX show that this was more stable in the cultures and did not

**Figure 4** Response profiles for melanoma cell lines treated with increasing concentrations of 7OH-MTX.

Figure 5 (a) Time course of uptake of [$^3$H]-MTX into B10 melanoma cells *in vitro*. Cells were incubated with a constant amount of [$^3$H]-labelled drug and unlabelled MTX added to the concentrations shown. ●—● $10^{-6}$ M MTX, ○—○ $10^{-5}$ M MTX. **(b)** Decreased uptake of [$^3$H]-MTX ($10^{-7}$ M) by B8 melanoma cells *in vitro* in the presence of 7OH-MTX. ●—● Control (no 7OH-MTX), ○—○ $10^{-6}$ M 7OH-MTX, ■—■ $10^{-4}$ M 7OH-MTX.

**Table I** Summary of HPLC analyses of MTX and 7OH-MTX metabolism *in vitro* by different cell lines

| *Line* | *MTX* | *7OH-MTX* |
|---|---|---|
| Medium alone | No change | No change |
| *Murine melanoma* | | |
| PG19 | Altered | Altered |
| *Choriocarcinoma* | | |
| BeWo | No change | Altered |
| JaR | No change | No change |
| *Human melanoma* | | |
| ADLER | No change | Altered |
| MYJ15 | No change | Altered |
| KOT | No change | Altered |
| B8 | Altered | No change |
| B10 | No change | No change |
| MEL57 | No change | No change |

HPLC and culture methods are described in the text yield the same metabolite profiles as MTX. Representative chromatograms obtained at 7 days from PG19 cells which metabolise both MTX and 7OH-MTX, and from B10 cells which metabolise neither compound are shown in Figure 6. In each experiment chromatograms from "control" cultures, incubated for the same time intervals in the absence of MTX and 7OH-MTX showed only the peak marked "M". In each case the baseline was flat in the area of the metabolite peaks, at a detector sensitivity of 0.08 AUFS. Supplementation of culture extracts with MTX or 7OH-MTX immediately prior to HPLC gave peaks eluting in the region of the profile assigned to the pure compound.

*Survival following leucovorin-rescue of cells* in vitro

Figure 7 shows that leucovorin rescues melanoma

**Figure 6** **(a)** HPLC profiles obtained from culture medium 11 days after incubation of PG19 cells in the presence of $10^{-6}$M MTX or 7OH-MTX. M = substance present in medium, A,B, = unknown metabolites. Position of methotrexate and 7OH-MTX shown dotted. Control cultures showed only peak M.

**Figure 6** **(b)** HPLC profiles obtained from culture media 11 days after incubation of B10 cells in the presence of $10^{-6}$M MTX or 7OH-MTX. M = substance present in medium. Position of MTX and 7OH-MTX shown dotted. Control cultures showed only peak M.

cells from MTX toxicity *in vitro*. The B10 cells resume growth following the addition of leucovorin to the culture medium 24 h after the application of $10^{-6}$M MTX. For comparison a choriocarcinoma cell, JaR, was included as an example of a cell line from a tumour known to be sensitive to MTX *in vivo*. The *in vitro* rescue by leucovorin of the choriocarcinoma resembles the B10 melanoma though an earlier response is apparent.

A differential response was observed by titrating MTX-inhibited cultures with increasing concentrations of leucovorin. Figure 8 shows that the choriocarcinoma JaR and B8 melanoma were rescued by leucovorin and gave similar profiles. We

**Figure 7** Rescue by leucovorin of human melanoma and choriocarcinoma cells from toxicity of MTX *in vitro*. ●—● B10 melanoma plus leucovorin, ○—○ B10 melanoma minus leucovorin, ■—■ JaR choriocarcinoma plus leucovorin, □—□ JaR choriocarcinoma minus leucovorin. The *in vitro* rescue assay was used. Cells were exposed to $10^{-6}$M MTX for 24h and toxicity reversed in the cultures indicated (plus leucovorin). The remaining cultures were put through the same rescue procedure but no leucovorin was added (minus leucovorin).

**Figure 8** Enhanced rescue by increasing concentrations of leucovorin. ●——● B8 melanona, ○——○ JaR choriocarcinoma, ▲——▲ KM3 (ALL). The *in vitro* rescue method was used. Cells were exposed to $10^{-6}$M MTX for 24h. Toxicity was reversed and incubation continues for 7 days in the presence of leucovorin as shown on the abscissa.

did not find leucovorin-rescue using the BeWo choriocarcinoma. KM3 (ALL) cells showed a rescue profile similar to JaR and B8 cells. The critical nature of the molar ratio of MTX to leucovorin *in vitro* is illustrated by an experiment (data not shown) in which rescue by the standard leucovorin method was ineffective for B10 cells when the concentration of MTX was raised to $5 \times 10^{-5}$M from the concentration of $10^{-6}$M used in the experiments shown in Figure 7.

### Discussion

This is the first study of melanoma patients treated with MTX in which plasma concentrations of the drug have been accurately monitored and the accumulation of metabolities observed and recorded. The results of these studies demonstrate that the lack of response of melanoma to MTX therapy is not due to failure to achieve adequate plasma concentrations. The concentrations achieved in our patients compared well with those known to be effective in patients with other MTX-sensitive tumours (see Breithaupt *et al.*, 1982). In previous clinical studies of MTX therapy in melanoma, total doses of $2\,gm^{-2}$ of MTX over 6h were used (Karakousis & Carlson, 1979) rather than 1.5g over 24h in our study. It is likely therefore that in these earlier studies even higher plasma concentrations may have been achieved. Inadequate plasma concentrations of MTX cannot therefore be the cause of the failure of MTX therapy to kill melanoma cells *in vivo*.

We have also shown that 2 melanoma cell lines studied (B8 and B10) take up MTX from the culture medium and that there is competition for MTX uptake by 7OH-MTX (Figures 5a and 5b). This finding suggests that the presence of 7OH-MTX could lead to reduced toxicity of MTX at later stages of an infusion and thus reduce the time for which the cells are exposed to adequate concentrations of MTX. However, the concentrations of 7OH-MTX found *in vivo* in plasma did not exceed those of MTX until 8 h after the start of the infusion by which time the intracellular concentration of MTX would be expected to be high.

Studies of the *in vitro* metabolism of MTX showed variable results depending upon the cell line investigated. Cultures of ADLER, MYJ15, KOT, B10 and MEL57 consistently failed to show metabolism of MTX in the medium, whereas cultures of B8 and PG19 metabolised the compound. However, the substances subsequently identified in the medium did not correspond on chromatographic analysis to any of the known MTX metabolites so far identified *in vivo*. In particular they were not MTX polyglutamates, which are known to be the major intracellular metabolites (Galivan, 1979). There was no correlation between those melanoma cell lines which metabolised MTX and resistance or sensitivity to it.

We have shown (Figure 2) that MTX arrests cell division at a concentration of $\sim 10^{-8}$ M and that following exposure to $10^{-6}$ M methotrexate for 24 h there was no recovery of cell division up to 11 days after removal of MTX (Figure 7).

We have also demonstrated that leucovorin can rescue melanoma cells in culture from the toxicity of MTX. The concentrations used in our *in vitro* experiments correspond to those reported for murine ascitic tumours by Sirotnak *et al.* (1978). These values might be expected to be found *in vivo* using a standard regime, from which it is clear that both melanoma and non-malignant cells could benefit from leucovorin-rescue. The fact that the choriocarcinoma JaR also responds to leucovorin-rescue *in vitro* indicates that tetrahydrofolate-rescue is not unique to melanoma and therefore probably not the sole reason for the failure of MTX/leucovorin therapy in patients with melanoma. However KM3 (ALL) cells sensitive to MTX *in vivo* showed considerable time dependency in that they failed to respond to leucovorin-rescue following 8 h of exposure to MTX.

The failure to rescue KM3 (ALL) cells in our standard assay may be related to a more rapid cell cycle than the melanoma lines used. Clinically this would account for a larger fractional cell-kill before the concentration of 7OH-MTX reached sufficient magnitude to interfere with cell-kill.

Our studies demonstrate competition by the major *in vivo* metabolite, 7OH-MTX, for the uptake of MTX by melanoma *in vitro*. If this is mirrored *in vivo*, after 12 h further uptake of MTX from the plasma would be inhibited by the higher concentrations of 7OH-MTX. In conjunction with the response to leucovorin-rescue, the effect would be to expose melanoma cells to MTX for a much shorter period than suspected hitherto. This may go some way towards explaining why these particular tumours are non-responsive to the drug using current therapeutic protocols.

In summary we have shown that the non-response of melanoma cells *in vivo* is unlikely to be related to the plasma MTX concentration, or to the ability of these cells to metabolise it. The plasma profile and *in vitro* data suggest that after 12 h at least two factors may contribute to the continued survival of melanoma cells: the concentrations of 7OH-MTX in plasma may be sufficient to inhibit the further uptake of MTX by these cells; secondly, leucovorin may rescue both viable melanoma cells and host tissue cells when current protocols are used. The additional possibility that melanoma *in vivo* is afforded protection from MTX cytotoxicity by as yet unidentified cell kinetic or biochemical mechanisms, such as the presence of salvaging metabolites (nucleosides or folates) remains to be investigated.

This work was supported in part by CRC Grant No SP 1382 P2. Y. Y. Farid is in receipt of a Postgraduate Scholarship from the Government of Iraq. We are grateful to Ms P.J. Price for typing the manuscript.

### References

BOSTOCK, C.K., CLARKE, E.M. HARDING, N.G.L. & 4 others (1979). The development of resistance to methorexate in a mouse melanoma cell line. *Chromosome*, **74**, 153.

BREITHAUPT, H., KUENZLEN, E. & GOEBEL, G. (1982). Rapid high pressure liquid chromatographic determination of methotrexate and its metabolities 7–hydroxymethotrexate and 2,4-diamino-N10-methylpteroic acid in biological fluids. *Annal. Biochem.*, **121**, 103.

CREASEY, A.A., SMITH, H.S., HACKETT, A.J. & 3 others (1979). Biological properties of human melanoma cells in culture. *In Vitro*, **15**, 342.

FARID, Y.Y.Z., WATSON, I.D. & STEWART, M.J. (1983). An assay for methotrexate and its metabolites in serum and urine by ion-pair chromatography. *J. Pharmacol. Biomed. Anal.*, **1**, (In press).

FISHER, R.I., CHABNER, B.A. & MYERS, C.E.(1979). Phase II Study of high dose methotrexate in patients with advanced malignant melanoma. *Cancer Treat. Rep.*, **63**, 47.

GALIVAN, J. (1979). Transport and metabolism of methotrexate in normal and resistant cultured rat hepatoma cells. *Cancer Res.*, **39**, 735.

JOHNS, D.G. & LOO, T.L. (1967). Metabolite of 4-amino 4-deoxy $N^{10}$-methylpteroylglutamic acid (methotrexate). *J. Pharmacol. Sci.*, **56**, 356.

KARAKOUSIS, C.P. & CARLSON, M. (1979). High dose methotrexate in malignant melanoma. *Cancer Treat. Rep.*, **63**, 1405.

SCHNEIDER, W., SCHWENK, H. & BURNKAMM, G. (1977). Characterisation of EBV genome negative null and T-cell lines derived from children with acute lymphoblastic leukaemia and leukaemic transformed non-Hodgkins lymphoma. *Int. J. Cancer*, **19**, 621.

SIROTNAK, F.M., MOCCIO, D.M. & DORICK, D.M. (1978). Optimisation of high dose methotrexate with leucovorin-rescue in L1210 leukaemia and sarcoma 180 murine tumour models. *Cancer Res.*, **38**, 345.

SMITH, S.A. & POGSON, C.I. (1980). The metabolism of tryptophan by isolated rat liver cells. *Biochem. J.*, **186**, 977.